# Varietal and Geographical Origin Characterization of Peaches and Nectarines by Combining Analytical Techniques and Statistical Approach

**DOI:** 10.3390/molecules26144128

**Published:** 2021-07-07

**Authors:** Gabriella Tamasi, Claudia Bonechi, Gemma Leone, Marco Andreassi, Marco Consumi, Paola Sangiorgio, Alessandra Verardi, Claudio Rossi, Agnese Magnani

**Affiliations:** 1 Department of Biotechnology, Chemistry and Pharmacy, University of Siena, Via Aldo Moro, 2, 53100 Siena, Italy; claudia.bonechi@unisi.it (C.B.); gemma.leone@unisi.it (G.L.); marco.andreassi@unisi.it (M.A.); claudio.rossi@unisi.it (C.R.); agnese.magnani@unisi.it (A.M.); 2 Centre for Colloid and Surface Science (CSGI), University of Florence, Via della Lastruccia 3, Sesto Fiorentino, 50019 Firenze, Italy; 3 National Interuniversity Consortium of Materials Science and Technology (INSTM), Via G. Giusti 9, 50121 Firenze, Italy; 4 ENEA, Trisaia Research Center, Italian National Agency for New Technologies, Energy and Sustainable Economic Development, Department of Sustainability, SS Jonica 106, km 419+500, 7026 Rotondella, Italy; paola.sangiorgio@enea.it (P.S.); alessandra.verardi@enea.it (A.V.); 5 Operative Unit, University of Siena, Campo Verde, Castrovillari, 87012 Cosenza, Italy

**Keywords:** *Prunus persica* L., vegetable matrix, reology, thermogravimetryic analysis, HPLC-ESI-MS, NMR spectroscopy, time of flight secondary ions mass spectrometry ToF-SIMS, antioxidants capacity, chemometry

## Abstract

*Prunus persica* L. is one of the most important fruit crops in European production, after grapes, apples, oranges and watermelons. Most varieties are rich in secondary metabolites, showing antioxidant properties for human health. The purpose of this study was to develop a chemical analysis methodology, which involves the use of different analytical-instrumental techniques to deepen the knowledge related to the profile of metabolites present in selected cultivars of peaches and nectarines cultivated in the Mediterranean area (Southern Italy). The comparative study was conducted by choosing yellow-fleshed peaches (*RomeStar*, *ZeeLady*) and yellow-fleshed nectarines (*Nectaross*, *Venus*) from two geographical areas (Piana di Sibari and Piana di Metaponto), and by determining the chemical parameters for the flesh and skin that allow for identification of any distinctive varietal and/or geographical characteristics. A combined analytical and chemometric approach was used, trough rheological, thermogravimetric (TGA), chromatographic (HPLC-ESI-MS), spectroscopic (UV-Vis, ATR-FTIR, NMR) and spectrometric (ToF-SIMS) analysis. This approach allowed us to identify the characterizing parameters for the analysis of a plant matrix so that the developed methodology could define an easily exportable and extendable model for the characterization of other types of vegetable matrices.

## 1. Introduction

Peach (*Prunus persica* L.), originated in China more than 4000 years ago [1] and has been cultivated in the areas of the Mediterranean Basin by the Roman Empire. Today, peaches and nectarines are among the most important fruit crops in Europe, after grape, apple, orange and watermelon productions. At the world level, with an annual world production of over 25.7 million tons, China contributes for 61.5% of the total production, followed remotely by Spain (6.0%), and then Italy (4.8%) on the basis of FAOSTAT database referring to 2019 data (last update December 2020 [2]). Peaches and nectarines are also the fruits with the highest number of new cultivars released per year from breeding programs in Europe, USA and China [3,4]. This huge production can be related not only to their pleasing characteristic flavor, but also to a better understanding of health benefits arising from the consumption of vegetables, and particularly polyphenolic rich fruits that are suggested for their ability to decrease the risk of chronic diseases such as cancer, diabetes and cardiovascular diseases [5,6,7,8]. Indeed, peach cultivars have been described as an excellent source of phytochemicals and natural antioxidants, showing a high antioxidant activity [9], and in-vivo study pointed out a negative correlation between peach polyphenolics and tumor growth and metastasis [10]. Selected polyphenols in peaches have been already identified, as well as the most abundant components, resulting in hydroxycinnamic derivatives, i.e., chlorogenic and neochlorogenic acids [11,12]. Peaches and nectarines are also rich in flavan-3-ols, flavonols, anthocyanins, quercetin and kaempferol derivatives [11]. However, cultivars can highly differ in their phenolic profiles and antioxidant properties, so studying the variation of the quality and quantity of bioactive components in commercial cultivated varieties [13,14,15] is crucial [16]. Nevertheless, the huge numbers of cultivars, the peaches, as well as the nectarines, similarity in morphological properties (color, size, shape), and the lack of a market classification, deeply affect the choices of consumers [17]. In addition, a continuous increase in consumer demand and expectations for quality products can be observed, so that producers and breeders aim to certify quality, authenticity and geographical origin of their products. Many producers take care to declare the agricultural practices (organic, biodynamic, etc.), as well as the practices related to the valorization of waste and by-products of agricultural and agro-industrial productions [18], underlining the actions towards a sustainable development and the reduction of greenhouse gases [19].

Several techniques and protocols have been developed and used to fully characterize the chemical profile of fruits. In particular, peach cultivars have been characterized by chromatographic methods such as column chromatography and high performance liquid chromatography coupled to electrospray ionization-mass spectrometry (HPLC-ESI-MS) [16] or to proton transfer reaction-mass spectrometry (PTR-MS) [17], which are highly sensitive techniques, but can require critical samples pretreatment. Mass spectrometry (MS) is one of the most sensitive chemical techniques for the analysis of natural products, owing to the virtue of its high sensitivity. Matrix-assisted laser desorption/ionization mass spectrometry (MALDI) and electrospray ionization (ESI) have been also successfully applied to the characterization of natural products [20,21].

Among the MS techniques, the Time of Flight Secondary Ions Mass Spectrometry (ToF-SIMS) allows for the direct analysis of solid samples, without requiring any sample pre-treatment, and allows for obtaining the chemical maps with a sub-micrometer spatial resolution. However, peach exocarps (fruit peels) have been mainly studied. This external layer, even if treated to remove any contaminants, can be deeply affected by the environmental condition and human manipulation.

The aim of this paper is the chemical characterization of different anatomical compartments of peaches and nectarines. The antioxidant capacity and phenolic profile of both exocarp (fruit peel) and mesocarp (flesh pulp) have been analytically determined. These data can represent a useful tool for authenticity control [22].

High resolution Nuclear Magnetic Resonance (NMR) has already been applied to discriminate the origin and variety of many fruits [23], identifying the most discriminating signals in the NMR spectrum, and to characterize the antioxidant compounds. In the present study, we report a detailed investigation of sugars and polyphenols content in peaches and nectarines by proton Nuclear Magnetic Resonance. Combining ^1^H NMR spectroscopy with principal component analysis (PCA), we also aim to identify some major compounds that are important for the discrimination of quality and geographical characterization.

Particular attention will be also paid to chemically characterize, via ToF-SIMS analysis, the seed integuments, which is protected by external contamination. The study will be carried out by comparing their chemical profile with those obtained from exocarp to find correlations with contamination and/or geographical origin. A collection of peaches and nectarines for their chemical profiles by ToF-SIMS, coupled to multivariate analysis, was investigated to analyze the difference in main components basing on cultivar and/or geographical location through the analysis of both exocarp (fruit peel) and seed integument.

The study was conducted by choosing, among the numerous cultivars that make up the varietal panorama present on the Italian market, four different cultivars (*Nectaross*, *Venus*, *RomeStar*, *ZeeLady*) and two geographical areas (Piana di Sibari main study area and Piana di Metaponto comparison area) for two reference varietal groups (yellow-fleshed peaches and yellow-fleshed nectarines) and by determining the chemical parameters to identify any distinctive varietal and/or geographical characteristics.

## 2. Results and Discussion

The peach and nectarine samples were chemically characterized analyzing ripeness parameter via rheological analyses, overall organic and inorganic composition via thermogravimetric analysis (TGA), total polyphenols (TPP) content and Trolox equivalent antioxidant capacity (TEAC) via spectrophotometric assays, and selected flavonoids components via chromatographic analysis (HPLC-ESI-MS). These analytical methods were coupled with semi-quantitative determination via ATR-FTIR, NMR spectroscopic methods, and ToF-SIMS analysis, with the aim of developing a multi-analytical methodology for peaches and nectarines, possibly extendable to other edible vegetable matrixes.

### 2.1. Ripeness Index via Rheological Analyses

Fruit ripening is an irreversible phenomenon involving biochemical and structural changes that lead to the development of a softer matrix [24]. As reviewed by Nambi and co-workers [25], several attempts were carried out to find a simple way to reveal the ripening stage of fruit analyzing different parameters, i.e., firmness, dry matter or total soluble solids [26], or fruit firmness and sugar/acid ratio [27]. Other studies tried to predict ripeness applying NIR spectroscopy [28]. Nevertheless, since ripening provokes strong changes in textural and rheological characteristics of fruit, the present study proposed combining rheology and TGA to gain information on the ripening stage of peaches. The fresh mesocarp samples were subjected to rheological analysis to establish the characteristics of the products in terms of viscoelastic properties and to allow a possible relation of these properties with the fruit ripening stage [29]. Elastic modulus (G’) and viscous modulus (G”) obtained for the samples (peaches and nectarines) were summarized in Table 1.

All samples showed a more predominant elastic rather than viscous behavior, comparable to results revealed on Mango fruits [30]. The parameter tan δ, being the ratio G’’/G’ could be considered a key value to gain information on the ripening stage of fruits. Indeed, it gives a measure of the relevance of the viscous component on the elastic one. The elastic component in a vegetal matrix can be related to the presence of a fibrous interconnected system. The stronger the interaction among fiber components in respect to soft components that during ripening increase, the lower tan δ.

Despite some differences in the values of their elastic and viscous moduli, samples show quite similar tan δ values, thus highlighting a similar soft component/hard component ratio and, as a consequence, a comparable ripening stage can be speculated.

### 2.2. Organic and Inorganic Composition via Thermogravimetric Analysis (TGA)

The fresh mesocarp samples underwent thermogravimetric analysis (TGA) to quantify the percentage composition in terms of aqueous content, sugars, fibers and minerals. The data are reported in Table 2.

Both peach and nectarine samples showed weight loss in the temperature range 30–200 °C, usually described as water content between 86–89% of fresh weight (fw). The weight loss in the temperature range 200–400 °C, which is reasonably attributable to the sugar contents, is also similar for all the samples (4–6% fw). Finally, the weight loss in the temperature range 400–600 °C, revealed a content of 1.4–2.1% of fibrous fraction. The residue after heating to 600 °C was about 3.7–5.8%, attributable to the mineral content.

TGA allowed us to have information on the ripening stage of fruit calculating the R parameter, defined as the ratio among the weight loss in 400–600 °C and 200–400 °C ranges [31]. As widely reported, labile organic matter (i.e., sugars, volatile compounds, linear polysaccharides) mainly decomposes in the 200–400 °C temperature range, whereas condensed components (fibers, strongly interacting vegetal matrixes) decompose in the 400–600 °C range [32]. The ripening process results in sugar accumulation and production of volatiles; meanwhile, a significant loss of firmness and cell walls disruption was observed [33]. Consequently, an increase of weight loss in the range of 200–400 °C was expected, as well as a reduction of weight loss in the following temperature range, i.e., 400–600 °C, as ripening proceeded. The higher the R value, the higher the fibrous contribution on weight loss. All samples showed no significant difference in calculated R values, suggesting a similar ripening stage (in perfect agreement with rheological studies, see above). Moreover, the ratio between the weight loss related to the entire organic components (200–600 °C) and the weight loss related to the water content (30–200 °C) was calculated, and this parameter was related to tan δ values, obtained by rheological analysis, and obtaining a strong linear correlation (r = −0.878; *p* < 0.01), confirming the reliability of these two techniques to obtain information on the ripening stage of fruit.

### 2.3. Antioxidant Properties: Spectrophotometric Analysis (TPP and TEAC) and HPLC-ESI-MS Analysis (Selected Flavonoids)

The antioxidant properties of the hydroalcoholic extracts (MeOH/H2O; 80:20%, *v*/*v*) of peach and nectarine mesocarp samples were evaluated trough two different spectrophotometric tests: (i) the Total Polyphenols content (TPP, via Folin–Ciocalteu assay) and (ii) the Trolox Equivalent Antioxidant Capacity (TEAC) based on the quenching of the ABTS^•+^ radical cation (Table 3). 

Regarding the TPP content, the experimental values were in the range 2416–4320 mg(GA)/kg dry weight (dw; equivalent to 676–1210 mg(GA)/kg fw, fresh weight, taking into account an average of 72% of water content in the analyzed fruits, see TGA data). These data revealed values usually slightly higher then values from literature: i.e., 281, 288–549, and 368–728 mg/kg fw in references [34,35,36], respectively. The TEAC values of the hydroalcoholic extracts from the peach and nectarine samples were in the range 9.4–29.2 mmol(Trx)/kg dw (Table 3; equivalent to 2.6–8.2 mmol(Trx)/kg fw, 72% of water content), in good agreement with TEAC values determined for the white-fleshed peaches samples (*Prunus persica* L. Batsch) from Sicily, 2.6–7.2 mmol(Trx)/kg fw [37]. On comparing the pairs of samples from the two different regions (Sibari and Metaponto Area) the TPP and TEAC parameters were statistically different for *Rome Star*, *Zee Lady* and *Nectaross* varieties. On the contrary, no significant differences (*p* > 0.05) were found for the *Venus* nectarine samples. It can be noticed that TPP and TEAC values for the commercial nectarine samples (Cx-Nx) averaged 2809 mg(GA)/kg dw and 13.68 mmol(Trx)/kg dw.

**Figure 1:**
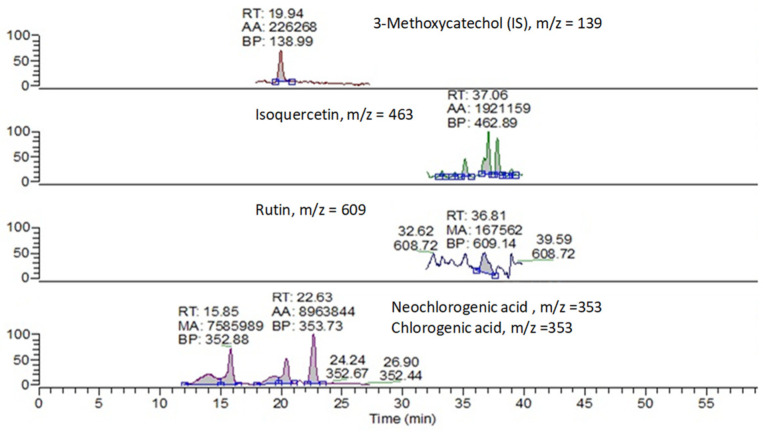
HPLC-ESI-MS chromatogram of hydroalcoholic extracts (MeOH/H_2_O; 80:20%, *v*/*v*) of sample 1S-VN.

The hydroalcoholic extracts (MeOH/H_2_O; 80:20%, *v*/*v*) of peach and nectarine mesocarp samples were also analyzed for the content of selected flavonoids (chlorogenic and neochlorogenic acids, quercetin glycoside derivatives (rutin and isoquercetin) and kaempferol) via liquid chromatography coupled with mass spectrometry. The chromatograms revealed, qualitatively, very similar patterns (Figure 1, sample 1S-VN).

**Figure 2:**
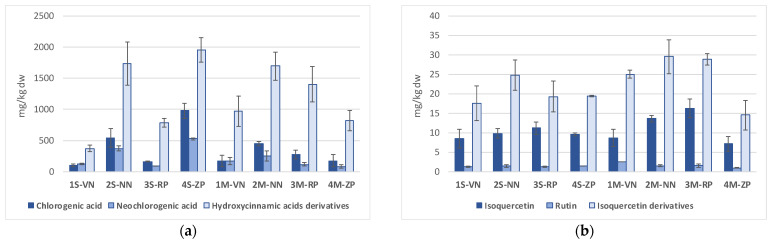
Histograms relevant to the contents of (**a**) chlorogenic acid (ChlAc), neochlorogenic acid (NeoChlAc), and hydroxycinnamic acids derivatives (HydcynDer) and (**b**) isoquercetin (IsoQue), isoquercetin derivatives (IsoQueDer), rutin (Rut) in hydroalcoholic extracts (MeOH/H_2_O; 80:20%, *v*/*v*) of peaches and nectarines mesocarp samples. The values are expressed as mean ± SD (*n* = 27; mg/kg dw).

The values found for the concentrations of the selected analytes are listed in Table 4, and reported in Figure 2, and they are in good agreement with those previously found and reported for peaches and nectarines mesocarps [12,38].

The data here reported for TPP, TEAC and selected quantified flavonoids were also treated and commented through a correlation matrix (Table 5) to emphasize the main relationships among parameters. Based on a comparative analysis of TEAC and TPP data, a positive linear correlation, characterized by r = 0.965 (*p* < 0.001) values, was obtained, in agreement with similar studies on peaches, apricots and plums [13,39].

In detail, the contents of chlorogenic and neochlorogenic acids were in the ranges 979–102 and 526–83 mg/kg dw. These values are also well comparable with values previously reported: 120–1820 and 50–1280 mg/kg dw [40], and are the major antioxidant components found in the present project. On comparing the contents of the selected antioxidants for nectarine samples (1S-VN, 2S-NN and 1M-VN, 2M-NN) the same trend was evident for the chlorogenic and neochlorogenic acids: the two *Venus* samples (1S-VN and 1M-VN) showed values not statistically different, but 4 to 5 times smaller than the corresponding values for the *Nectaross* samples (2S-NN and 2M-NN).

Instead, in regards to the peach samples (3S-RP, 4S-ZP and 3M-RP, 4M-ZP), the contents of the two hydroxycinnamic acids were much lower than those for the nectarines, excluding the case of sample 4S-ZP that showed the highest contents for both chlorogenic and neochlorogenic acids, being 979 and 526 mg/kg dw, respectively. The commercial sample of nectarines Cx-Nx showed data well comparable to those found for *Venus*, *RomeStar* and *ZeeLady* varieties. As regards the chlorogenic acids derivatives, even the presence of the cryptochlorogenic acid (4-*o*-caffeoylquinic acid) and other derivatives, like *p*-coumaroylquinic acid (337 *m*/*z*) are reported in literature [38]. A further peak at retention time R_t_ = 22.63 min (Figure 2) was detected, even in samples analyzed in the present study, in the region of the chlorogenic acids (353 *m*/*z*), and it was attributed to the 4-*o*-caffeoylquinic acid isomer and was quantified as chlorogenic acid equivalent (in absence of a reference standard).

The total content of the hydroxycinnamic acids derivatives was also determined (Table 4) and peach 4S-ZP and nectarine 2S-NN and 2M-NN samples revealed the highest concentrations of hydroxycinnamic acids when compared to the other samples. A trend similar to that found for the chlorogenic and neochlorogenic acids was revealed by the correlation matrix (Table 5): chlorogenic acid, neochlorogenic acid and their derivatives showed a significant correlation from strong (*p* < 0.05) to very strong (*p* < 0.01) among themselves and with TPP and TEAC, the strongest being chlorogenic acid vs. neochlorogenic acid (r = 0.963; *p* < 0.001).

Regarding the contents of selected quercetin glucosides, isoquercetin and rutin showed values in the range 16.3–7.1, and 2.6–1.0 mg/kg dw, respectively (Table 4, Figure 2; that are equivalent to 4.6–2.0 and 0.6–0.28 mg/kg fw, considering the 72% of water content). These latter compounds are present in a lower amount with respect to the hydroxycinnamic acids, just reported and discussed, revealing values barely comparable with the few data recovered from literature: i.e., total content of isoquercetin and rutin in Western Red nectarines 0.25 mg/kg fw [38]. The highest contents for quercetin glucosides derivatives were reported for the skin portions of yellow flesh variety peaches and nectarines, up to 74.1 mg/kg fw for total flavonols [12]. The data obtained in the varieties analyzed in present work (Table 4, Figure 2) showed that both isoquercetin and rutin are equally distributed in all samples, and 2M-NN and 3M-RS showed the highest values for isoquercetin (13.6 and 16.3 mg/kg dw, respectively). Given the possible presence of further glucoside derivatives of quercetin at the same *m*/*z* value (i.e., quercetin-3-galactoside or even further isomers of isoquercetin), the total values of isoquercetin derivatives were quantified as isoquercetin equivalents (Table 4). A distribution pattern similar to that observed for isoquercetin is evident, showing contents in the range 31.5–14.5 mg/kg dw, slightly lower than values previously reported in literature (38.7 mg/kg dw, taking into account 90% water content [12]). Correlation matrix (Table 5) highlighted a strong significant correlation between isoquercetin and its derivatives (r = 0.708, *p* < 0.05) and a moderate not significant correlation with rutin (r = 0.516, *p* > 0.05). It is interesting to note that sample 1M-VN showed an outlier behavior, and excluding it from the data, the correlation between flavonols increases, becoming significant (isoquercetin *vs* derivatives: r = 0.765, *p* < 0.05; isoquercetin *vs* rutin: r = 0.767, *p* < 0.05).

Finally, regarding kaempferol, its content was much lower than that of other investigated components, and just reported, being in the range 0.45–0.11 mg/kg dw (Table 4, Figure 2), showing differences often not statistically significant, among the varieties/geographical origins.

### 2.4. Infrared Analysis (ATR-FT-MIR)

The FT-MIR spectra were recorded through an ATR technique on lyophilized powdered mesocarp and lyophilized external skin of peaches and nectarine samples. All the samples were analyzed in triplicate and the skin samples were selected for analyzing both red and yellow areas.

**Figure 3:**
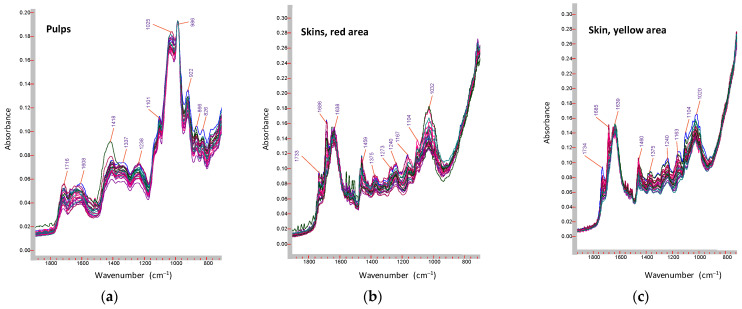
Superimposition for ATR-FT-MIR spectra relevant to lyophilized samples in the spectral region 1900–700 cm^−1^: (**a**) mesocarp (pulp), (**b**) skin, red area, and (**c**) skin, yellow area.

All samples showed superimposable MIR spectra for mesocarps and skins (red and yellow area), as reported in Figure 3. Typical bands of main components were revealed. In particular, amide I and amide II bands, characteristics for the protein peptide bonds, can be observed at 1650 cm^–1^ (amidic C=O stretching) and 1550 cm^–1^ (amidic NH bending). The stretching vibration bands of the protonated (–COOH) and deprotonated (–COO^–^) carboxyl groups from organic acids are also present at 1718 and 1610 cm^–1^. Finally, the spectral region between 1200–900 cm^–1^ is characterized by the sugar bands (sucrose, glucose and fructose) and by the bands that comes from vibrations of organic acids molecules (mostly malic and citric acids).

Particularly, these bands are assigned to C–C and C–O stretching, as also previously found on apricots mesocarp (pulps [41]).

Nevertheless, comparing skin spectra with mesocarp ones, significant differences in the regions 1800–1500 and 1200–800 cm^−1^ were revealed (Figure 3). Indeed, all skin samples spectra showed a high intensity of the bands in the region 1800–1500 cm^−1^, specifically C = C stretching vibrations of aromatic systems, 1620–1590 cm^−1^ and OH bending vibrations, ca. 1640 cm^–1^, due to cyanidin and cyanidin derivatives that are mainly located in the exocarp. Contrarily, mesocarp samples, that are richer than skin in sugars, showed MIR spectra characterized by high intensity of the bands of sugar moieties (1200–800 cm^−1^).

### 2.5. H NMR Analysis

**Figure 4:**
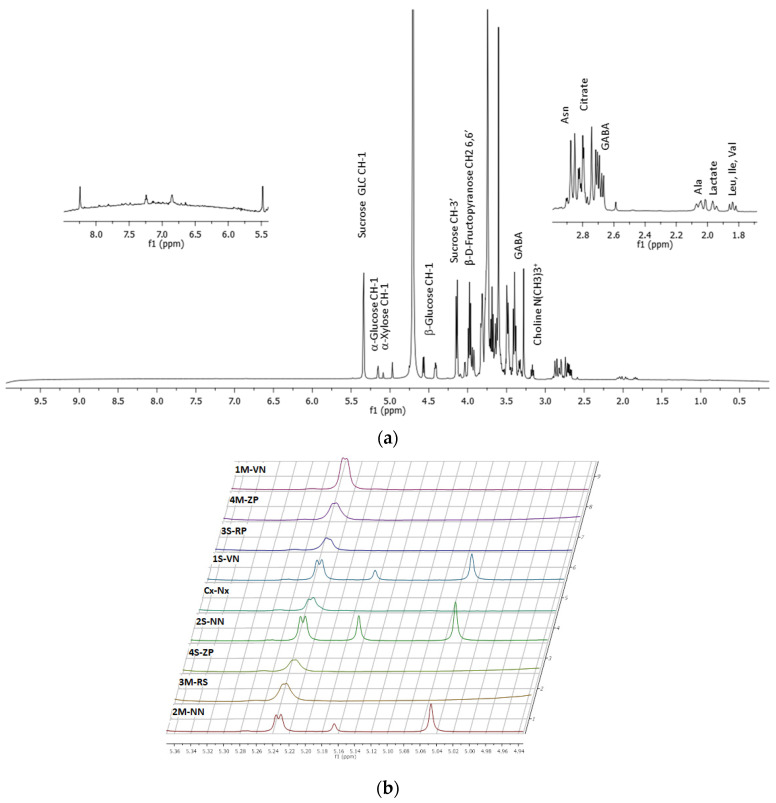
(**a**) ^1^H-NMR spectrum of Sibari Area-*Venus*/Nectarine (sample 1S-VN) recorded at 600 MHz and 298 K. Two ^1^H-NMR expansions in the ranges 5.5–8.5 ppm, and 1.5–3.0 ppm are also reported. (**b**) Staking plot of ^1^H-NMR spectra of all the samples, in the region 4.70–5.60 ppm.

To identify the principal proton NMR signals present in different extract and to highlight differences between samples, NMR spectra of lyophilized peaches in D_2_O were recorded at 600 MHz and 298 K. The ^1^H-NMR spectrum of sample 1S-VN (Sibari Area-*Venus*/Nectarine), recorded at 600 MHz, was reported in Figure 4a.

Previous studies reported that in the peach and nectarine it is possible to identify different components, such as organic acids (citric acid, malic acid, fumaric acid, succinic acid, quinic acid, shikimic acid), sugars (glucose, fructose, sucrose and xylose), and several amino acids (alanine, valine, threonine, asparagine, isoleucine, phenylalanine and GABA) [42,43,44]. In particular, it was possible to identify some of the major water-soluble metabolites: for the sucrose GLC CH-1 at 5.37 ppm and the CH-3’ at 4.14 ppm; for the α-glucose CH-1 at 5.15 ppm; for the β-glucose CH-1 at 4.57 ppm; for the α-xylose CH-1 at 5.08 ppm and the β-d-fructopyranose CH-2-6,6’ at 3.93 ppm. At 2.74 and 2.85 ppm the β, β’-CH2 protons of asparagine were also identified. The methyl protons (N(CH_3_)_3_^+^) of choline show a signal at 3.16 ppm [43]. In the 5.5–9.0 ppm region, NMR signals due to the polyphenols and flavonoids compound were showed [44].

The stacking plots of proton NMR spectra for all samples, in the region 4.70–5.60 ppm, were also reported in Figure 4b. Samples 1S-VN, 2S-NN, and 2M-NN showed peaks at 5.37 ppm (sucrose), 5.15 ppm (α-glucose), 5.08 ppm (CH-1 α-xylose) and 4.98 ppm that are not present in other spectra. These experimental data suggest that the sugar composition in these samples could be different. This behavior was revealed also in other region of NMR spectra, such as a variation of the chemical shift in the region between 4.6 and 4.4 ppm, due to sugar component (CH-1 of the β-xylose and CH-1 of the β-fucose). Specifically, it was evident that the samples 1S-VN, 2S-NN, and 2M-NN exhibited a very intense singlet at 8.3 ppm, not present in the spectra recorded for other samples.

**Figure 5:**
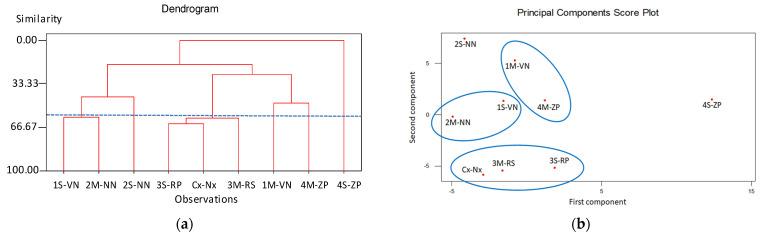
Principal Component Analysis (PCA) and cluster analysis from ^1^H NMR data applied to score plots for all the samples: (**a**) Agglomerative Hierarchical Clustering (AHC) dendrogram for the correlation of variables; (**b**) results for 60% of similarity.

To examine the metabolic variability combined with multivariate statistical analysis, the proton NMR spectra were acquired for all samples. The integrals of all normalized NMR signals (assigned and not assigned) were therefore calculated. To perform the statistical analysis, an appropriate pre-treatment of the data was carried out with the autoscaling method, focusing the attention on the chemical information by reducing the noise associated with the measurement. These data were used to build the experimental data matrix for Principal Components Analysis (PCA) approach. The result of PCA score plot is reported in Figure 5a. The PC1 and PC2 components were responsible for about 65% of the total variance. In particular, the first three components (PC1, PC2 and PC3) represent the 35.54, 29.65 and 17.84% of the data variability, respectively.

The PCA results suggest that all samples were similar. Only for the sample 4S-ZP, located along the positive value of PC1, a behavior different from the others can be highlighted, so much so that it can be defined as an outlier. From the *loading plot* it was revealed that the variables were distributed in all directions, and just the metabolites C20 (proton NMR signal at 4.41 ppm) and C21 (sucrose CH-3′ at 4.14 ppm) were distributed along the positive axis of the first principal component and they are responsible for the behavior of the sample 4S-ZP. This result suggests the reducing of the number of variables for the PCA analysis (from 76 at 11). In particular, the variables related to the sugar component (α-glucose, β-glucose, sucrose, β-d-fructopyranose, β-d-fructofuranose, β-xylose), amino acids (isoleucine, threonine, valine) and chlorogenic acid, represent the most important parameters that influence the variance of the samples.

Hierarchical clustering analysis was used to arrange the samples into dendrogram based on their similarity. In the dendrogram (Figure 5a), the proximity of two connected samples represents the similarity of their NMR profiles. If the spectral data dendrogram is truncated at a relative distance of 60% of similarity, three clusters are obtained (Figure 5b). Based on the individual sample characteristics, it was revealed that clusters A and B contain three samples, respectively, and clusters C contains only two samples. Hierarchical clustering analysis confirmed that sample 2S-NN and 4S-ZP are outliers. The cluster does not include samples from the same geographic location.

The PCA analysis does not reveal any the correlation between samples and geographic origin, but the clusters showed a correlation between nectarine varieties (samples 1S-VN and 2M-NN) and peach varieties (samples 3S-RP and 3M-RS). The commercial nectarine sample (Cx-Nx) was included in the cluster with samples 3S-RP and 3M-RS. Only the cluster with samples 1M-VN and 4M-ZP showed a correlation between two different varieties (Figure 5b). The multivariate statistical analysis allows to establish strong correlations between the different varieties of peaches and nectarines, identifying clusters that combine both nectarines and peaches from different geographical origins.

### 2.6. ToF-SIMS Analysis

**Figure 6:**
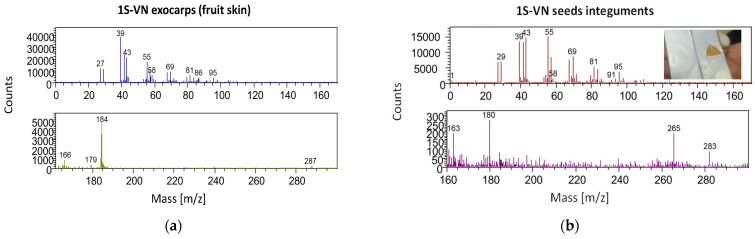
ToF-SIMS positive ion spectra of (**a**) exocarp (fruit skin) and (**b**) seed integument of sample 1S-VN.

The negative ions ToF-SIMS spectra of exocarps (fruit skin) and seeds integuments, in particular in the high mass region, provided very few relevant information. Thus, only the positive ion spectra are reported and analyzed. Moreover, positive ions spectra can give information on relative abundance of metal ions on samples that can help in geographical characterization of samples. In the low mass region (0–150 *m*/*z*) typical peaks obtained from fragmentation of esters and alcohols derivatives were found in both exocarp (fruit skin) and seed integument spectra (Figure 6) as also previously identified [17]. Differently, acetic acid and methanol peaks cannot be found in significant amount.

Some significant differences were revealed in the high mass region (150–300 *m*/*z*) in both exocarps and seed integuments spectra. As regards the exocarps (fruits skin, Figure 6a) samples, the most significant peaks were centered at 179.06 *m*/*z* (C_9_H_7_O_4_^+^, cyanidin fragment), 184.06 *m*/*z* (C_5_H_14_PO_4_N^+^, phosphatidyl choline fragment) and 287.15 *m*/*z* (C_15_H_11_O_6_^+^, cyanidin molecular ion). On the other side, the spectra of seed integuments samples showed the same peaks, but with a very significant lower intensity. On the contrary, intense peaks of coniferyl alcohol (180.00 *m*/*z*, C_10_H_12_O_3_^+^, molecular ion; 163.00 *m*/*z*, C_10_H_11_O_2_^+^, coniferyl alcohol fragment) and oleic acid (283.00 *m*/*z*, C_18_H_35_O_2_^+^, molecular ion; 265.00 *m*/*z*, C_18_H_34_O^+^, oleic acid fragment) were revealed as highlighted in the spectrum of 1S-VN seed integument sample (Figure 6b). Thus, confirming NMR and ATR-FT-MIR results. No significant differences were found in terms of metal ions distribution.

PCA analysis was applied to ToF-SIMS data. As previously reported, all the peaks, that were at least three times the background in the 0–400 *m*/*z* region, were considered to build a matrix for all of the spectra from all of the samples. This resulted in a list of 529 and 758 peaks for the positive ion spectra of seed integument and exocarp (fruit skin), respectively. The inclusion of all the peaks minimized the BIAS, introduced into the analysis by the peak selection. A combination matrix of all seeds integuments and exocarps peaks was produced and that resulted in a list of 878 parameters. A clear separation of seeds integuments and exocarps was revealed.

**Figure 7:**
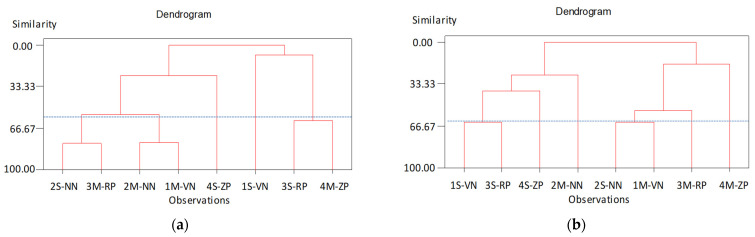
Agglomerative Hierarchical Clustering (AHC) dendrogram from ToF-SIMS positive ion spectra, on: (**a**) seeds integuments samples and (**b**) exocarps (fruit skins) samples.

The cluster analysis was also performed on seeds integuments and exocarps samples separately. Interestingly, a different result was obtained. In particular, the cluster analysis on seeds integuments samples clearly distinguished peaches and nectarines harvested in Sibari from those harvested in Metaponto area (Figure 7a), whereas cluster analysis on exocarps (fruit skins) did not show any clear trend, using 1.3 of dissimilarity as cut-off (Figure 7b). This result can be related to the fact that the seeds integuments is certainly less subjected to pollution by external/environmental factors that can alter its chemical composition of the fruits and particularly of the exocarp (skin). Ultimately, and therefore, the cluster analysis obtained from the seed integuments is certainly a more viable path for the geographical characterization of peaches and nectarines than that carried out on the fruits exocarp.

## 3. Conclusions

The study developed a method of chemical analysis planned for a full characterization of vegetal products, for human nutrition. In particular, the study was carried out on samples of yellow-fleshed peaches (*Prunus persica* L. Batsch) and yellow flesh nectarines (*Prunus persica* L. Batsch, var. Nectarina) of four different cultivars (*RomeStar*, *ZeeLady*, peaches; *Venus*, *Nectaross*, nectarines) from two geographic areas of Southern Italy, the Sibari Area and the Metaponto Area, located on the Ionian coast of Calabria and Basilicata, respectively. A further sample of commercial nectarine was considered with unknown origin and cultivars. These were chemically characterized by different and complementary analytical techniques, to identify and quantify the chemical composition, with particular efforts to key phytochemical components.

1. Thermo-gravimetric (TGA) and rheological analyses were very useful to characterize the ripening stage of the sample, revealing a great homogeneity among them.
2. The determinations of the antioxidant capacity (TEAC method, quenching the ABTS^•+^ radical cation) and total polyphenols (Folin–Ciocalteu method) of mesocarp (flesh pulp) hydroalcoholic extracts revealed as both peaches and nectarines are excellent sources of natural antioxidant polyphenols.
3. HPLC-ESI-MS analysis on mesocarp (flesh pulp) hydroalcoholic extracts, allowed the identification and quantification of selected polyphenolic compounds, revealing a predominant relative distribution of the two chlorogenic and neochlorogenic acids and hydroxycinnamic acids derivatives, with a statistically significant linear relation with findings for antioxidant activity and total polyphenols.
4. ^1^H-NMR spectra revealed the presence of sugars (sucrose, *α*- and *β*-glucose, *α*-xylose, *β*-d-fructopyranose) among the main constituents of mesocarp extracts, presenting only minor differences in chemical shift and peaks intensity between samples, in accordance with a chemical composition very similar between peaches and nectarines. In particular, the samples of nectarine variety *Venus* and *Nectaross* showed additional peaks at 5.16 ppm and 5.05 ppm due to a different sugar composition (presence of *α*-xylose) and at 8.30 ppm in the aromatic signal region. The PCA analysis obtained by statistical processing of the ^1^H-NMR spectra, showed the presence of two outlier samples (*ZeeLady*-Peach and *Nectaross*-Nectarine, 2S-NN and 4S-ZP) in accordance with different chemical compositions observed in the high values of chlorogenic acid and neochlorogenic acid from chromatographic measurements. Furthermore, statistical Cluster analysis showed the grouping of samples for variety, between two samples of nectarines (one *Nectaross* and the other *Venus*) and between two samples of peaches (both *RomeStar*) with a significance level of 60%.
5. In agreement with NMR data, IR measurements carried out on lyophilized samples of mesocarps (pulps) and exocarps (skins), confirmed the presence of characteristic bands of –COOH groups of organic acids, –OH groups of sugars, phenols, water, and peptide groups (NH–CO) (amide bands I, II and III) of the proteins. From the comparison between the IR spectra of skin with those of flesh pulp it was established that more intense absorption bands in the region 1800–1500 cm^–1^ of the exocarp are attributable to cyanidins, while the more intense absorption bands corresponding to sugars between 1200–800 cm^–1^ are obtained from the mesocarp.
6. ToF-SIMS analysis confirmed the presence of cyanidin and phosphatidylcholine in the exocarps of peaches and nectarines, and cyanidin, phosphatidylcholine, oleic acid and coniferyl alcohol in the seed integuments. The cluster analysis obtained from the seed integuments constitutes a viable tool for the geographical characterization of peaches and nectarines. Furthermore, the PCA analysis (and cluster analysis), performed on seed integuments ToF-SIMS data, found two varietal grouping for nectarines and peaches and the presence of two outliers.

## 4. Materials and Methods

### 4.1. Chemicals

All reactants and analytical standards were purchased from Sigma-Aldrich (Milan, Italy), and are hereafter listed: Folin–Ciocalteu’s phenol reagent, sodium carbonate (Na_2_CO_3_, ≥ 99.5%), gallic acid (GA; 3,4,5-trihydroxybenzoic acid, ≥ 99%), potassium persulfate (K_2_S_2_O_8_, ≥98%), ABTS (2,2’-azino-bis(3-ethylbenzthiazoline-6-sulphonic acid, ≥98%), trolox (Trx; 6-hydroxy-2,5,7,8-tetramethylchroman-2-carboxylic acid, ≥97%), chlorogenic acid (ChlAc, 3-*o*-caffeylquinic acid, ≥95.0%), neochlorogenic acid (NeoChlAc, 5-*o*-caffeylquinic acid, ≥98%), quercetin (Que, ≥95%); isoquercetin (IsoQue, quercetin-3-*o*-β-d-glucoside, ≥90%), rutin trihydrate (Rut, quercetin-3-*o*-rutinoside, ≥95%), kaempferol (Kaemp, ≥90%), 3-methoxycatecol (MeOCat, ≥99%; IS, internal standard for HPLC-MS determinations). All solvents were HPLC grade 99.9% (Sigma-Aldrich, Milan, Italy): methanol (MeOH), ethanol (EtOH), acetonitrile (AcCN), formic acid (HCOOH, 98%), acetic acid (CH_3_COOH; 98%). The bidistilled water was produced by an Acquinity P/7 distiller (MembraPure GmbH, Berlin, Germany). Deuterated solvents: D_2_O (99.9% D), dimethylsulfoxide D6 (99.8% D), methanol D4 (99.8% D, H_2_O < 0.03%) were purchased from VWR Prolabo (Milan, Italy) and tetramethylsilane (TMS, 99.7%) from Merck (Milan, Italy).

### 4.2. Sample Collection and Pre-Treatment

**Figure 8:**
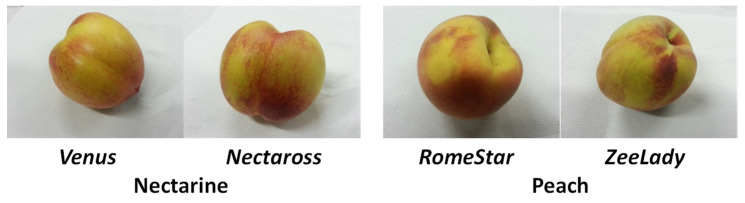
Varieties of peaches and nectarines analyzed in the present study.

The fruits analyzed in this study were chosen between yellow-fleshed peaches (*Prunus persica,* L. Batsch) and yellow-fleshed nectarines (*Prunus persica,* L. Batsch, var. Nectarine), belonging to the *Rosaceae* family. In particular, four different cultivars were analyzed (*Venus, Nectaross, Rome Star and Zee Lady*, Table 6; Figure 8) from two different geographical areas of Southern Italy: Sibari Area (Calabria region) and Metaponto Area (Basilicata region). In addition, a sample of commercial nectarine (Cx-Nx, Commercial/Nectarine) of unknown geographical origin and unknown variety was chosen for comparison reasons. The samples were collected at maturity stage (in triplicate, to average the biological variability) and treated within 48 h from the picking.

The mesocarp (pulp; from three fruits for each sample type) was homogenized through a kitchen-mixer (3 min at max speed; Moulinex DPA141, Moulinex International, Ecully Cedex, France) and freeze-dried. The pulps were weighed before and after lyophilization to determine the moisture content (Radwag AS 220/C/2; max capacity, 220 g, readability, 0.0001 g). Lyophilized samples were then powdered in an agata mortar and stored in polyethylene containers, at −2 ± 1 °C, before subsequent analyses (spectrophotometric assays, HPLC-ESI-MS and NMR), to preserve the polyphenols content and antioxidant properties.

The exocarps of the fruits (peel,) and the seeds integuments were also collected. The exocarps (peel, about 0.5 mm) and seeds integuments were removed using a stainless-steel blade, and compressed between two glazing slides, lined with an aluminum foil, to obtain flat and smooth surfaces and to avoid any external contamination. The samples were suitably lyophilized, and then stored at −32 ± 1 °C, before subsequent ATR-FTIR and ToF-SIMS analyses.

### 4.3. Rheology

Rheological analyses were carried out on fresh slices of mesocarp samples (pulp about 5 mm thick, in triplicate) by using a controlled strain rheometer AR-2000 (TA Instruments). A plate–plate geometry impermeable to fluid flow (diameter, 40 mm; gap between plates, 1 mm) was used. Measurements were performed at 25.0 ± 0.5 °C. The frequency sweep test was performed to obtain *G*′ and *G*″ parameters, as a function of frequency (0.1–10 Hz) imposing 2% strain, obtained by preliminary strain sweep tests [45].

### 4.4. Thermogravimetric Analysis (TGA)

Thermogravimetric analyses (TGA) were carried out using a SDT Q-600 thermogravimeter (TA Instruments). Aliquots of 15 mg of each fresh mesocarp samples (pulp) were analyzed (in triplicate), applying a thermal program from 30 to 600 °Cwith a heating ramp of 10 °C/min, under constant nitrogen flow, 100 mL/min.

### 4.5. Antioxidant Hydrofilic Componets Extraction Procedure

The extraction protocol was optimized based on procedures previously published [12,13], with some modifications. Briefly, aliquots of 1.00 g of lyophilized peach/nectarine mesocarps (pulps, analytically weighted) underwent solid/liquid extraction procedure by 20 mL of MeOH/H_2_O (80:20%, *v*/*v*) ultrasound assisted (10 min, at 25 ± 2 °C; power, 120 Watt; sound frequency, 35 kHz; ultrasonic bath Sonorex Bandelin), and subsequent centrifugation (5 min, 4000 rpm; Thermo Electron Corporation PK 110). The solid residue was treated two more times by 10 mL solvent mixture (each time), to allow a quantitative recovery, and the liquid fractions were added each to others (final volume, 40 mL). The extracts were directly used (diluted if necessary) for antioxidant assays (total polyphenols and TEAC determinations). Amounts of 5 mL were dried under ultrapure N_2_ flow, freeze-dried, and finally stored in the dark at −32 ± 1 °C, before HPLC-ESI-MS and NMR measurements. All the samples were extracted in triplicate.

### 4.6. Antioxidant Activity Assays

#### 4.6.1. Total Polyphenols (TPP) Content: Folin–Ciocalteu Assay

Total polyphenol contents (TPP) were determined via the common colorimetric method using the Folin–Ciocalteu reactant [46,47], with some modifications [18,48]. Briefly, aliquots of 500 µL of fresh filtered extracts (syringe PTFE filter; porosity, 0.20 µm) were 10-times diluted and treated adding 500 µL Folin–Ciocalteu reactant and 2 mL of Na_2_CO_3_ (20%, p/v aqueous solution), up to a final volume of 10 mL water. The treated samples were then mixed and incubated (30 min, 21 ± 2 °C, in the dark). Finally, the absorbance at 750 nm (Abs_750_) was recorded trough a dual-beam (against water) Perkin Elmer Lambda EZ 201 spectrophotometer (Monza, Italy), equipped with software PESSW 1.2 (spectral range, 190–1100 nm; optical pathway, 10 mm; cuvettes, PMMA/UV grade). The calibration curves were recorded by using gallic acid standard solutions (range 0.25–10.00 mg/L) and calibrations showing correlation factors R^2^ > 0.990 were accepted for analyses. The results were expressed as mg of gallic acid equivalents per kg of dried samples (mg(GAE)/kg dw). Each extract was treated in triplicates and each replicate was analyzed in triplicates.

#### 4.6.2. Trolox Equivalent Antioxidant Capacity (TEAC) Assays

The antioxidant capacity was evaluated on the basis of the TEAC spectrophotometric tests based on the quenching of the ABTS^•+^ radical cation, following procedures previously reported [47,49,50], with some modifications [18,48]. A stable stock solution of ABTS^•+^ was prepared by reacting a water solution of ABTS (7 mM) and K_2_S_2_O_8_ (140 mM) and letting the mixture incubating (12–16 h, 10 ± 1 °C, in the dark). The dark blue-green formed radical solution was then properly diluted in EtOH (just before the use) to obtain an absorbance of 0.70 ± 0.02 at 734 nm (blank; see above for spectrophotometer details). A volume of 1.0 mL of ABTS^•+^ alcoholic solution was then treated with a known volume of extract (properly diluted, if necessary) and incubated (30 min, 21 ± 2 °C, in the dark), before to record the absorbance at 734 nm (Abs_734_; against EtOH). The calibration curves were recorded by using Trolox standard solutions (range 0.20–20.0 µM) and calibrations showing correlation factors R^2^ > 0.990 were accepted for analyses. The calibration curves were plotted as ∆A_734_% *vs* Trolox standard concentrations, ∆A_734_% being the relative decreasing in absorbance of radical solutions treated with standards or samples, with respect to the blank solution (ABTS^•+^ not treated):

∆A_734_% = {[1 − (A_734(Trolox/Sample)_/A_734(Blank)_)] × 100} (1)

The results were expressed as mmol Trolox equivalent per kg of dried sample, (mmol(Trx)/kg dw). Each extract was treated in triplicates and each replicate was analyzed in triplicates.

### 4.7. HPLC-ESI-MS Analysis

Chromatograms were acquired through HPLC-ESI-MS instrument (Agilent Technologies 1200 Series HPLC, MS Thermo-Scientific TSQ Quantum Access), managed by the Xcalibur software (Thermo-Scientific), following methods previously published [18,48,51] with some modification. The chromatographic separation was carried out by a C18 reverse phase column (Phenomenex Luna; 250 × 4.6 mm; 5 μm particles, 100 Å porosity) with a Phenomenex SecurityGuard pre-column (Phenomenex C18, 4.0 × 3.0 mm), thermostated to 30 °C. The mobile phases were: (A) water acidified with acetic acid (H_2_O/CH_3_COOH, 0.1%) and (B) methanol and the elution was as here following reported: in linear gradient: from 0 to 5.0 min, 80% A (isocratic); from 5.0 to 35 min: 80–50% A (linear); from 35 to 37 min, 50–20% A (linear); from 37 to 45 min: 20% A (isocratic); from 45 to 47 min, 20–10% A (linear); from 47 to 48 min, 10–80% A (linear); from 48 to 60 min, 80% A (isocratic); at a flow rate of 0.70 mL/min, splitting the flow by a 2:1 splitter, before the introduction to the ion source. The injection volume was 20 μL.

The ESI-MS conditions were optimized via direct injection of quercetin in MeOH, in negative ionization mode (spray voltage 4000 V, sheath gas and auxiliary gas pressure 60 and 30 arbitrary units, capillary temperature 270 °C). Experiments were carried out in Selected Ion Monitoring (SIM) mode, selecting the *m*/*z* of the target analytes and confirming their identity in the samples by comparison of their retention times with those of the standard compounds reported in Table 7.

The main parameters relevant to the analytical determinations and quantification of chlorogenic acid (ChlAc), neochlorogenic acid (NeoChlAc), kaempferol (Kaemp), isoquercetin (IsoQue) and rutin (Rut) are also reported in Table 7. The quantifications were carried out via external calibration method, using 3-methoxycatecol as internal standard (IS). Stock standard solutions were freshly prepared (10 mg/10 mL of MeOH) and then diluted to the desired concentrations. The stock solutions were stored at −20 ± 1 °C for a maximum of 48 h. Calibration curves were acquired by injection of standard solutions in MeOH/H_2_O (80/20%, *v*/*v*) in the range of linearity as reported in Table 7. Calibrations showing correlation factors *R^2^* > 0.980 were accepted for analyses, and limits of detection (LOD) and limits of quantification (LOQ) values were also evaluated (Table 7). To assess the precision of the quantification method, intra-day and inter-day calibrations were carried out (at three different concentrations, *n* = 3), revealing relative standard deviation percentage (% RSD) in terms of normalized peak area, lower than 4%.

The dried extracts (see above) were re-dissolved in 2 mL of MeOH/H_2_O (80/20%, *v*/*v*) solvent and filtered by syringe PTFE filter (porosity, 0.20 µm) before injection. Each standard/extract was injected in triplicate.

### 4.8. Infrared Analysis (ATR-FTIR)

Lyophilized pulp (mesocarp) samples and the external site of lyophilized exocarps (skins) were analyzed via an FT-IR instrument (Agilent Technologies Cary 630, ZnSe engine) equipped with an attenuated total reflection (ATR) accessory, a diamond crystal as internal reflection element and a Mercury/Cadmium/Tellurium (MCT) detector. The spectra were recorded in the range 4000–750 cm^–1^, trough 128 averaged scans (resolution, 2.0 cm^–1^). Baseline and spectra were performed using the MicroLab FTIR Agilent software and were treated by the Varian Resolutions software (v. 4.1.0.101).

### 4.9. ^1^H-NMR Spectroscopy

All samples for the NMR analysis were prepared, loading 500 μL of the D_2_O at the lyophilized product (100 mg). ^1^H-NMR spectra were acquired at 298 K on a Bruker DRX-600 AVANCE spectrometer, equipped with an *xyz* gradient unit and operating at 600.13 MHz. The data was processed with Bruker XWINNMR Software, version 2 on Silicon Graphics equipped with RISC R5000 processor, working under the IRIX 6.3 operating system.

### 4.10. Time-of-Flight Secondary Ion Mass Spectrometry (ToF-SIMS)

The ToF-SIMS analyses were performed on lyophilized exocarp of the fruits and seed integuments, by a TRIFT III time of flight secondary ion mass spectrometer (Physical Electronics, Chanhassen, MN, USA) equipped with a gold liquid-metal primary ion source. The lyophilized exocarps and seeds integuments were positioned overnight in a conditioning pre-chamber (vacuum value of 10^−4^ Pa) and then moved to the analyzing chamber (vacuum value to 10^−8^ Pa). Positive and negative ions spectra were acquired using a 22 keV Au^+^ primary ion beam which was rastered over a (100 × 100) µm area. Static SIMS conditions (primary ion dose density < 1012 ions/cm^2^) were maintained. Selected peaks were used to calibrate positive and negative ions spectra, in the low mass region: positive CH_3_^+^ (15.023 *m*/*z*), C_2_H_3_^+^ (27.023 *m*/*z*), C_3_H_5_^+^ (41.039 *m*/*z*); and negative CH^−^ (13.008 *m*/*z*), OH^−^ (17.003 *m*/*z*), C_2_H^−^ (25.008 *m/z*). A number of peaks of increasing mass were assigned and added to the calibration set for an accurate mass calibration. The mass resolution (*m*/*m*) was 6000 at 27 *m*/*z*.

### 4.11. Statistical Data Analysis

All the samples were pre-treated (extracted and lyophilized) in triplicate, and all the analyses were also performed in triplicate. All the analytical data were reported as mean values ± standard deviations (SD; *n* = 27). Analysis of variance (ANOVA) followed by Tukey’s post-hoc test was performed to determine significant differences and data showing *p* values < 0.05 were considered statistically significant (GraphPad Prism version 5.04, GraphPad Software Inc., San Diego, CA). Selected parameters were also analyzed for linear regression and Pearson correlation matrix (*p* value at 95% confidence interval) [52].

The ^1^H-NMR data were treated via a chemometric multivariate approach, though PCA and cluster analysis models (Agglomerative Hierarchical Clustering, AHC) that allowed untargeted analysis to explore varietal and/or geographical origin characterization. The spectra treatment was performed by using NCSS 2004 Statistical Analysis System Software, to help the identification of the biochemical parameters distributions. All the spectra were treated normalizing the integrals of all the signals (assigned and non-assigned), they were autoscaled and then, used to build the experimental data matrix. This matrix undergoes PCA analysis and only PCA with eigen values > 1, explaining more than a single parameter alone, were used. The same data were treated by cluster analysis approach.

The ToF-SIMS data were treated via a chemometric multivariate approach, though PCA and cluster analysis models (Agglomerative Hierarchical Clustering, AHC). Prior to multivariate analysis, all the peaks that were at least three times the background in the 100–400 *m*/*z* region were considered to create a table for all of the spectra from all of the samples. The peaks were then normalized to their total intensity to correct for differences in total secondary ion yield from spectrum to spectrum, which can result from instrumental drift. The clusters analysis was determined from the dendrogram by defining a dissimilarity cut-off (i.e., a vertical line positioned at 2/3 of the maximum relative distance). In this paper, cluster analysis was carried out by defining the distance between samples based on euclidean distance (Ward’s method). Cluster analysis was performed using Past3 software [53].

## Figures and Tables

**Table 1:** G’, G” and tan δ (i.e., G”/G’) parameters from rheological analysis on fresh slices of peaches and nectarine mesocarp.

Samples	G’ (kPa) (1 Hz)	G” (kPa) (1 Hz)	tan δ
1S-VN	85 ± 6	11 ± 2	0.129
2S-NN	138 ± 12	15 ± 2	0.109
3S-RP	173 ± 8	18 ± 3	0.104
4S-ZP	121 ± 9	10 ± 1	0.083
1M-VN	180 ± 8	18 ± 4	0.100
2M-NN	138 ± 10	14 ± 3	0.101
3M-RP	81 ± 9	11 ± 2	0.135
4M-ZP	24 ± 1	2.0 ± 0.1	0.083

**Table 2:** TGA analysis of fresh peach and nectarine mesocarp samples. Values expressed as weight loss percentage (%; instrumental sensitivity, 0.5%). R parameter, ratio among the weight loss in 400–600 °C and 200–400 °C ranges, is also reported.

Samples	30–120 °C	120–200 °C	200–400 °C	400–600 °C	Residue	R
1S-VN	78	10.7	4.0	1.4	5.8	0.35
2S-NN	68	20.3	5.6	1.6	5.1	0.28
3S-RP	82	7.5	5.1	1.8	3.7	0.35
4S-ZP	76	10.4	6.5	2.1	5.1	0.32
1M-VN	77	10.5	6.0	1.7	5.1	0.28
2M-NN	60	27.4	5.3	1.8	5.4	0.34
3M-RP	69	20.3	4.6	1.6	4.6	0.35
4M-ZP	67	21.4	5.8	1.7	4.2	0.29

**Table 3:** Values of Total Polyphenols (TPP, mg(GA)/kg dw) and Trolox Equivalent Antioxidant Capacity (TEAC, mmol(Trx)/kg dw) of hydroalcoholic extracts (MeOH/H_2_O; 80:20%, *v*/*v*) of peaches and nectarine mesocarp samples. The values are expressed as mean ± SD (*n* = 27). Values marked with the same letter within the same column are not statistically different (Tukey’s test, *p* > 0.05).

Samples	TPP (mg(GA)/kg dw)	TEAC (mmol(Trx)/kg dw)
1S-VN	2416 ± 36 ^a^	9.44 ± 0.41 ^a^
2S-NN	3326 ± 38 ^b,c^	17.56 ± 0.20 ^b^
3S-RP	2702 ± 327 ^a,d^	11.36 ± 0.23 ^c^
4S-ZP	4320 ± 130 ^e^	29.21 ± 2.41 ^d^
1M-VN	2573 ± 291 ^a,d^	9.76 ± 0.01 ^a^
2M-NN	3723 ± 661 ^f^	18.61 ± 0.82 ^e^
3M-RP	3591 ± 351 ^c,f^	23.34 ± 0.70 ^f^
4M-ZP	3043 ± 554 ^b,g^	16.56 ± 1.05 ^g^
Cx-Nx	2809 ± 444 ^d,g^	13.68 ± 0.34 ^h^

**Table 4:** Contents of chlorogenic acid (ChlAc), neochlorogenic acid (NeoChlAc), hydroxycinnamic acids derivatives (HydcynDer), isoquercetin (IsoQue), isoquercetin derivatives (IsoQueDer), rutin (Rut) and kaempferol (Kaemp) in hydroalcoholic extracts (MeOH/H_2_O; 80:20%, *v*/*v*) of peaches and nectarines mesocarp samples. The values are expressed as mean ± SD (*n* = 27; mg/kg dw). Values marked with the same letter within the same column (for each analyte) are not statistically different (Tukey’s test, *p* > 0.05).

**Samples**	**ChlAc (mg/kg dw)**	**NeoChlAc (mg/kg dw)**	**HydcynDer (mg/kg dw)**	
1S-VN	102 ± 19 ^a^	123 ± 10 ^a,b^	372 ± 56 ^a^	
2S-NN	540 ± 155 ^b^	368 ± 41 ^c^	1732 ± 346 ^b^	
3S-RP	164 ± 1 ^a,c^	89 ± 3 ^a,d^	788 ± 79 ^c^	
4S-ZP	979 ± 120 ^d^	526 ± 16 ^e^	1949 ± 195 ^d^	
1M-VN	169 ± 92 ^a,c^	171 ± 56 ^f^	968 ± 242 ^e^	
2M-NN	445 ± 36 ^e^	255 ± 83 ^g^	1694 ± 223 ^b^	
3M-RP	275 ± 64 ^f^	117 ± 33 ^a,b,d^	1402 ± 280 ^f^	
4M-ZP	176 ± 10 ^c^	83 ± 23 ^d^	819 ± 164 ^c,e^	
**Samples**	**IsoQue (mg/kg dw)**	**IsoQueDer (mg/kg dw)**	**Rut (mg/kg dw)**	**Kaemp (mg/kg dw)**
1S-VN	8.5 ± 2.4 ^a,b^	17.5 ± 4.4 ^a^	1.21 ± 0.18 ^a^	0.27 ± 0.16 ^a^
2S-NN	9.8 ± 1.2 ^b,c^	24.7 ± 3.9 ^b^	1.39 ± 0.34 ^a,b,c^	0.45 ± 0.04 ^b^
3S-RP	11.2 ± 1.6 ^c^	19.3 ± 4.0 ^a^	1.30 ± 0.22 ^a,b^	0.17 ± 0.04 ^c^
4S-ZP	9.6 ± 0.4 ^b^	19.4 ± 0.2 ^a^	1.36 ± 0.01 ^a,b^	0.16 ± 0.04 ^c,d^
1M-VN	8.7 ± 2.2 ^b^	25.0 ± 1.0 ^b^	2.55 ± 0.01 ^d^	0.25 ± 0.04 ^a^
2M-NN	13.6 ± 0.8 ^d^	29.5 ± 4.3 ^c^	1.46 ± 0.26 ^b,c,e^	0.26 ± 0.01 ^a^
3M-RP	16.3 ± 2.4 ^e^	28.9 ± 1.5 ^c^	1.59 ± 0.47 ^c,e^	0.16 ± 0.05 ^c,d^
4M-ZP	7.1 ± 2.0 ^a^	14.5 ± 3.8 ^d^	0.95 ± 0.09 ^f^	0.11 ± 0.01 ^d^

**Table 5:** Correlation matrix for quantified analytical parameters (TPP, TEAC, and selected quantified flavonoids: chlorogenic acid (ChlAc), neochlorogenic acid (NeoChlAc), hydroxycinnamic acids derivatives (HydcynDer), isoquercetin (IsoQue), isoquercetin derivatives (IsoQueDer), rutin (Rut), kaempferol (Kaemp)).

	TPP	TEAC	ChlAc	NeoChlAc	HydcynDer	IsoQue	IsoQueDer	Rut
TEAC	0.965 ***							
ChlAc	0.882 **	0.834 **						
NeoChlAc	0.749 *	0.679 *	0.963 ***					
HydcynDer	0.890 **	0.798 **	0.855 **	0.803 **				
IsoQue	0.385	0.366	0.054	−0.074	0.332			
IsoQueDer	0.165	0.095	−0.002	0.022	0.204	0.708 *		
Rut	−0.207	−0.249	−0.131	−0.010	0.007	0.112	0.516	
Kaemp	−0.072	−0.190	0.148	0.354	0.292	−0.080	0.204	0.155

*** *p* < 0.001; ** *p* < 0.01; * *p* < 0.05; parameters without stars showed *p* > 0.05.

**Table 6:** Geographical origin and variety of samples analyzed in the present study.

Sample	Origin	Variety
1S-VN	Sibari Area	*Venus* (Nectarine)
2S-NN	Sibari Area	*Nectaross* (Nectarine)
3S-RP	Sibari Area	*Rome Star* (Peach)
4S-ZP	Sibari Area	*Zee Lady* (Peach)
1M-VN	Metaponto Area	*Venus* (Nectarine)
2M-NN	Metaponto Area	*Nectaross* (Nectarine)
3M-RP	Metaponto Area	*Rome Star* (Peach)
4M-ZP	Metaponto Area	*Zee Lady* (Peach)
Cx-Nx	Commercial	(Nectarine)

**Table 7:** Selected parameters for HPLC–ESI-MS and calibration method for hydroxycinnamic acids (chlorogenic acid, ChlAc; neochlorogenic acid, NeoChlAc) and flavonoids (isoquercetin, IsoQue; rutin, Rut; kaempferol, Kaemp) identification and quantification. Parameters for the internal standard (IS; methoxycatecol, MeOCat) are also reported. (SIM, Single ion monitoring: LOD, limits of detection; LOQ, limits of quantification).

	t_R_ (min)	MS Mode Polarity	[M−H]^−^ (*m*/*z*)	Calibration Range (mg/L)	Equation R^2^	LOQ // LOD (mg/L)
ChlAc	20.65	SIM negative	353	0.010–15	y = 8.40731 × 0.9995	0.010 // 0.003
NeoChlAc	15.85	SIM negative	353	0.010–15	y = 9.00526 × 0.9998	0.010 // 0.003
IsoQue	37.06	SIM negative	463	0.010–10	y = 16.06550 × 0.9952	0.010 // 0.003
Rut	36.81	SIM negative	609	0.010–12	y = 14.10963 × 0.9969	0.010 // 0.003
Kaemp	41.02	SIM negative	285	0.060–1.0	y = 11.22166 × 0.9924	0.060 // 0.020
MeOCat	19.94	---	139	IS, 0.5	---	---

As an example, typical equations and R^2^ values, relevant to the calibration carried out for a set of measurements, are also reported. Hydroxycinnamic acid derivatives and isoquercetin derivatives were quantified as chlorogenic acid equivalent and isoquercetin equivalent, respectively.

## Data Availability

Data sharing not applicable.

## Sample Availability

Samples are not available.
